# Ecological Momentary Intervention for Memory in Chronic Traumatic Brain Injury: Findings From a Mixed-Methods Pilot and Feasibility Study

**DOI:** 10.1097/HTR.0000000000001147

**Published:** 2026-01-29

**Authors:** Emily L. Morrow, Lyndsay A. Nelson, Melissa C. Duff, Lindsay S. Mayberry

**Affiliations:** **Author Affiliations:** Division of General Internal Medicine & Public Health, Department of Medicine, Vanderbilt University Medical Center, Nashville, Tennessee (Drs Morrow, Nelson, and Mayberry) and Department of Hearing & Speech Sciences, Vanderbilt University Medical Center, Nashville, Tennessee (Dr Duff).

**Keywords:** ecological momentary assessment, memory, mobile health, text messaging, traumatic brain injury

## Abstract

**Objective:**

To examine acceptability and feasibility of a technology-delivered memory assessment and intervention tool (MEMI, memory ecological momentary intervention) and its intended delivery schedule for adults with chronic traumatic brain injury (TBI). MEMI introduces new information, cues retrieval, and assesses learning via text messages.

**Setting:**

Community sample from Vanderbilt Brain Injury Patient Registry in Nashville, TN, USA.

**Participants:**

20 adults with chronic, moderate-severe TBI and 20 non-injured comparison (NC) participants matched on age, sex, and education.

**Design:**

In this crossover study, participants used MEMI for two weeks: one in which they completed all learning in a single session to approximate standard memory rehabilitation (Blocked schedule) and one in which retrievals were spaced in short, twice-daily sessions (Spaced, intended delivery schedule).

**Main Measure(s):**

The primary pilot outcomes were feasibility (session engagement) and acceptability (Acceptability of Intervention Measure). In a preliminary analysis, we also assessed delayed free recall of trained information. We also report supplementary data on participant surveys and cued recall.

**Results:**

Participants in both groups were highly engaged and rated the tool as acceptable. On average, the TBI group rated acceptability of the Spaced schedule higher than the Blocked schedule. Both groups remembered more words at the end of the week in the Spaced schedule (TBI: *t*(17) = 4.37, *p* <.001, 95% CI [2.79, 7.99]; NC: *t*(19) = 8.57, *p* <.001, 95% CI[5.13, 8.46]).

**Conclusions:**

In this pilot study, spaced retrieval via MEMI was feasible and acceptable for adults with chronic TBI. MEMI has potential as a memory assessment and intervention tool in both therapeutic and research contexts. Future work should include a larger trial to assess MEMI’s efficacy, clinical utility, individual differences, and personalizing content.

TRAUMATIC BRAIN INJURY (TBI) is now understood as a chronic condition, rather than an acute injury with constrained recovery.^1-3^ Symptoms may persist for the rest of a person’s life.^1-5^ While adults with TBI face multiple challenges to life participation, memory deficits should be a key focus for chronic care because they are among the most common, damaging, and persistent symptoms.^4,6,7^ In fact, more than half of people with moderate-severe TBI face memory deficits,^3^ which affect participation at school and work^6-9^ and are detected 10 years after injury.^10^ Yet, memory rehabilitation is currently focused in the acute and subacute injury phases,^4,11,12^ and recent decades have seen limited progress in developing effective memory therapies.^6^

Memory is not a one-time event, but rather an iterative process occurring across multiple repeating phases (i.e., encoding, consolidation, retrieval).^13^ Critically, each retrieval of learned information is an opportunity to strengthen memory for that information through a process called reconsolidation.^14^ Deficits that limit participation in daily life may not be detected in a single assessment session, like a doctor’s appointment, single-session neuropsychological memory assessment, or therapy visit.^11,15^ Using multiple assessments across phases of memory, we have shown that adults with chronic TBI do not consolidate (strengthen, stabilize) their memories over time like non-injured peers.^16^ In a word learning study, adults with TBI retrieved fewer words than non-injured peers when tested immediately (i.e., after encoding), but the gap between the groups in the number of words retrieved grew over the course of a week.^16^ This increasing gap, which would not be captured by a typical single-session memory assessment, suggests a deficit in memory consolidation that is only identifiable by ongoing assessment over time.^9,16^

Memory disorders should be assessed and managed across contexts.^9,17^ Robust findings in the cognitive neuroscience literature indicate that adults, including those with brain damage, learn best in diversified contexts.^18-20^ This diversity may involve retrieving information at different times and places (i.e., spaced retrieval^21,22^), emulating the way people learn in the real world. Yet, memory therapy often occurs in a single context (i.e., same clinic room, same day of week, same time, same therapist). This represents a missed opportunity, as each memory retrieval is both an assessment and an opportunity to strengthen memory for the retrieved information through reconsolidation (i.e., an intervention).^14^

Technology-delivered interventions may move rehabilitation from the therapy room into daily contexts and extend beyond the acute injury phases, creating systems that support people with TBI in the real world and over the lifespan. Ecological momentary assessment and intervention frameworks leverage technology-delivered inquiries (e.g., via text message or push notifications from apps) to support individuals’ behavior in their daily lives.^23-26^ These frameworks may offer an opportunity to assess memory over time, help therapists and patients to identify memory patterns for a personalized approach to rehabilitation, and support memory for trained information via multiple retrieval and reconsolidation opportunities in diverse contexts.

We designed memory ecological momentary intervention (MEMI), a tool that uses text messaging to introduce new information, cue retrieval, and assess learning over time and context. Because repeat, technology-delivered memory rehabilitation represents a paradigm shift from existing practice, it was important to establish whether MEMI was feasible to deliver, usable, and acceptable for adults with TBI. In earlier work, we found that MEMI was highly usable and engaging for adults with TBI, and we used participant feedback to refine the tool.^27^ Here, we conducted a pilot study to assess MEMI’s feasibility, acceptability, and intended delivery schedule for a subsequent evaluative trial. We aimed to understand how using MEMI to space retrievals affects its feasibility and acceptability relative to standard rehabilitation schedules. Consistent with NIH pilot trial guidelines,^28^ these feasibility and acceptability analyses were our primary aims. We also conducted preliminary analyses to assess if spacing retrievals via MEMI has the predicted supportive effect on recall of trained information.

## METHODS

### MEMI functionality

**Figure 1.**
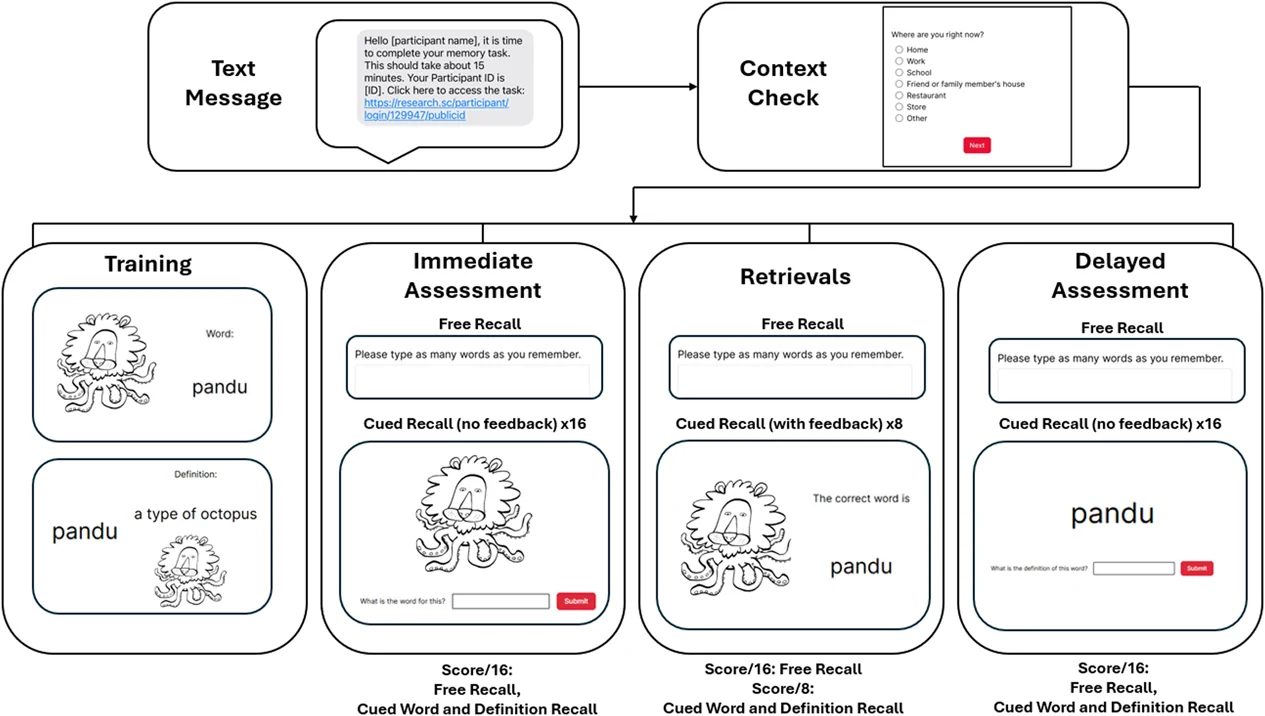
MEMI visuals. Assessments and retrievals include free recall and a mix of cued recall tasks assessing word and definition recall. Examples of possible cued recall tasks are given in each box, but these assessment formats are interspersed in each session. Only retrievals include feedback after participant response.

MEMI works via simple text messaging with a mobile web interface. Automated text messages are delivered to schedule using Twilio integrated with REDCap.^29^ Participants click on a link in the text message to complete memory tasks online using the Gorilla behavioral experiment platform.^30^ Participants can choose the timing of their text message prompts to work with their schedules. We briefly describe the memory tasks below and give examples in Figure 1.

#### Context checks

Before every MEMI session, participants answer a single question about their spatial context. This allows us to assess the number of spatial contexts experienced throughout the week, as well as changes in spatial context between training and assessment.

#### Training

Participants first complete a 15-minute training session in which they see each of 16 novel words (items) 4 times each. The items are presented visually to ensure that headphones are not required, and training is kept brief to limit participant burden.

#### Immediate and delayed assessments

Immediately after the training (Immediate), and again at the end of the week (Delayed), participants complete a 5-8-minute assessment. First, participants free type all the items they learned (i.e., free recall, the most robust indicator of learning^13^). Then, they respond to cues (e.g., seeing an image and providing the name for that image). The tasks are designed to tap into memory for both individual elements of an experience (e.g., the word form) and the relations between elements (e.g., mapping a word’s form to its meaning). For the Immediate and Delayed assessments, participants do not receive feedback on their responses. The goal of these assessments is to assess what memories participants form after the training (encoding, Immediate) and how those memories change over the course of the memory process (consolidation, Delayed).

#### Retrievals

Between the Immediate and Delayed Assessment, participants complete short retrievals of a subset of the trained items. Each retrieval includes 8 of the 16 trained items, and items are cycled so that each item is repeated equally. Participants complete a mix of the same free- and cued-recall tasks as the Assessments. However, in the Retrieval sessions, participants see the correct answer after responding to each cued recall item to ensure that they gain repeated exposure to the trained information. See the Design section for how retrieval schedules varied in this study.

#### Content

In this pilot trial, target items were novel word forms like the ones in Figure 1. However, we designed MEMI to flexibly deliver any target items, with the goal that the system will eventually be customizable for individual learning targets.

### Pilot trial

This research was approved by the Institutional Review Board at Vanderbilt University (IRB#221 667). The pilot trial ran from May 2024 to December 2024. All participants provided written informed consent.

#### Sample

The target sample was 20 adults with chronic, moderate-severe TBI and 20 non-injured comparison (NC) participants. Participants were recruited from the Vanderbilt Brain Injury Patient Registry.^31^ All participants with TBI were in the chronic phrase (>6 months post-injury^2,31^). TBI severity was moderate-severe per the Mayo Classification System.^32^ We chose to focus on the chronic phase of moderate-severe TBI because people with moderate-severe TBI are most likely to experience lasting memory deficits,^3,7^ and our eventual goal is to extend memory treatment to daily life in the chronic phase of injury. Participants were 18-55 years of age to exclude developmental effects of TBI and conservatively limit the effects of age-related cognitive decline. Participants with TBI had no other neurological or cognitive disabilities aside from the qualifying injury.

We also recruited NCs to understand how MEMI affects the TBI group’s performance relative to peers. NCs were also recruited from the Vanderbilt Brain Injury Patient Registry, had no history of neurological or cognitive disability, and were matched to the participants with TBI on age, sex, and education.

#### Recruitment

At the start of the pilot, we contacted a group of Registry participants with TBI that was selected to be diverse regarding sex, age, and education to garner a wide range of perspectives. Each participant received an email and had the opportunity to indicate interest or schedule a call to learn more about the study. When a participant with TBI enrolled in the study, their corresponding NC was then recruited.

#### Design

**Figure 2.**
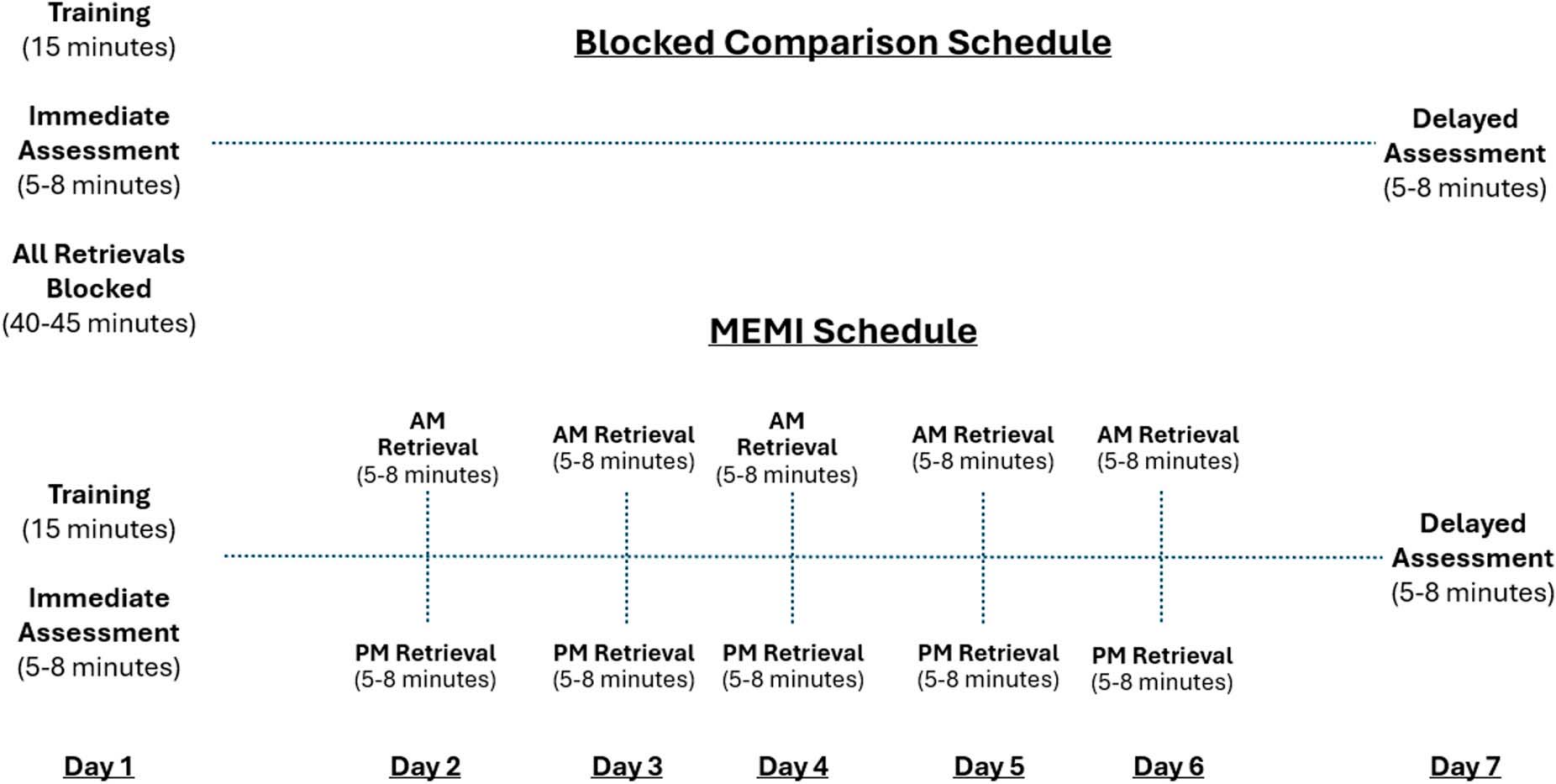
Condition schedules. All participants completed both schedules. The Blocked schedule was designed to emulate a traditional rehabilitation structure, with all learning in a single session and evaluation after one week. The Spaced schedule included interspersed retrievals throughout the week, and these sessions occurred at participants’ preferred morning and evening times.

In this crossover design, all participants completed both schedules (Figure 2). In the Blocked (comparison) schedule, participants completed the Training, Immediate Assessment, and all Retrievals in a single session at the beginning of the week, then did not receive any more texts until they completed the Delayed Assessment. This schedule was designed to emulate a standard therapy structure (e.g., weekly sessions in outpatient therapy). In the intended Spaced delivery schedule, participants completed the same number of retrievals in short, twice-daily sessions over the course of the week. The spacing of retrievals was uniform (i.e., the interval between retrievals did not increase over the course of the study). The order of schedules was counterbalanced. We also used two different word lists, which were counterbalanced across schedules. Having all participants complete both schedules allowed us to directly compare the two schedules within participants to ensure stability of individual characteristics (e.g., memory, digital literacy). The crossover design also allowed us to understand how adults with TBI responded to MEMI, and each schedule, relative to matched peers.

#### Technical support

At the beginning of the experiment, participants reviewed an instructional handout with an experimenter (Supplementary Digital Content 1, available at: http://links.lww.com/JHTR/B32). They completed the rest of the experiment on their own smartphones. Each participant had an assigned experimenter available for on-call technical support throughout the experiment.

#### Measures

##### Participant characteristics available from Brain Injury Patient Registry

Demographic and injury characteristics were collected from medical records and a semi structured interview when participants joined the Registry. These included age, sex, years of educational attainment, time since injury, and acute injury characteristics used to characterize severity. Registry participants completed two neuropsychological assessments of episodic memory: the Rey Auditory Verbal Learning Test^33^ and the NIH Toolbox Picture Sequence Memory Text^34^ (Supplementary Digital Content 3, available at: http://links.lww.com/JHTR/B32).

##### Digital literacy

We assessed digital literacy at the beginning of the study using an adapted version of the Digital Health Care Literacy Scale,^35^ a validated 3-item measure with scores ranging from 0 to 12 (higher = more digital health literacy). Because MEMI is delivered via text messages, we modified questions to focus on mobile phone use.

##### Everyday memory

We assessed subjective daily memory performance at the beginning of the study using the Everyday Memory Questionnaire,^36^ a validated 13-item measure of memory failures in everyday life with scores ranging from 0 to 5 (lower = fewer memory failures).

##### Feasibility

We quantified the number and proportion of available sessions completed by each participant. In the Blocked schedule, this included 2 sessions (1 Training/Initial Assessment/Retrieval and 1 Delayed Assessment). In the Spaced schedule, this included 12 sessions (1 Training/Initial Assessment, 10 Retrievals, and 1 Delayed Assessment). We did not prompt participants to complete sessions beyond the automated texts, and compensation did not depend on session engagement.

##### Acceptability

The primary acceptability measure was the Acceptability of Intervention^37^ measure, a 5-item self-report measure. Scores range from 4 to 20 (higher = better). Participants also responded to free text survey questions (Supplementary Digital Content 4, available at: http://links.lww.com/JHTR/B32).

##### Preliminary analysis of outcomes

The primary outcome for our preliminary analysis was free recall, which represents the most robust form of learning.^13^ Free recall was scored based on the number of exact target word forms that participants typed without cueing. Scores range from 0 to 16 (higher = better). See Supplementary Digital Content 5, available at: http://links.lww.com/JHTR/B32 for information on cued recall scores.

#### Analyses

We calculated descriptive statistics for participant characteristics, engagement, acceptability, and preliminary outcomes using R (version 4.2.1). For the preliminary outcomes analysis, we used paired-samples t-tests within each group to compare free recall at the Delayed Assessment between the two schedules. See Supplementary Digital Content 4-5, available at: http://links.lww.com/JHTR/B32, for analysis of free text survey responses and cued recall scores.

## RESULTS

### Retention

Participants who consented but withdrew before participating were replaced [41 consented (20 TBI, 21 NC) and 40 participated (20 TBI, 20 NC)]. Of the 40 included participants, 38 completed all study components (18 TBI, 20 NC). One participant with TBI withdrew after the first week (Spaced schedule) because he was unable to operate his smartphone after a software update. Another participant with TBI withdrew after his first week (Blocked schedule) because he grew frustrated with the word learning task. Otherwise, all participants completed the experiment. Therefore, Blocked analyses include 19 participants with TBI and 20 NCs, and Spaced analyses included 19 participants with TBI and 20 NCs.

### Sample characteristics

#### Demographic and injury characteristics

The TBI group was half female and had a mean age of 41.1 years (SD: 10.4) and mean educational attainment of 14.7 years (SD: 2.3). The NC group was also half female and had a mean age of 40.7 years (SD: 9.8) and a mean educational attainment of 14.6 years (SD: 2.3). The two groups did not differ significantly on age (*t*(39) = 0.13, *p* = 0.90) or education (*t*(39) = 0.07, *p* = 0.95). Mean time since injury in the TBI group was 8.1 years (SD: 6.2, range 2.1-22). Although this was not an exclusionary criterion, all participants in both groups were community-dwelling (i.e., did not live in a nursing home). Ninety percent (36/40) of the sample identified as Non-Hispanic White, one participant identified as Hispanic, one participant identified as Native American or Pacific Islander, and two participants identified as more than one race. See Supplementary Digital Content 2, available at: http://links.lww.com/JHTR/B32 for demographic and injury characteristics of the TBI group.

#### Digital health literacy

The sample’s self-reported digital literacy on the modified Digital Health Care Literacy Scale^35^ was moderately high, with an average score of 10.5 (SD: 1.9, range: 7-12) in the TBI group and 11.2 (SD: 1.4, range: 7-12) in the NC group, and did not differ between groups (*t*(38) = 1.31, *p* = 0.20).

#### Everyday memory

Participants with TBI reported more memory failures in daily life than NCs on the Everyday Memory Questionnaire^36^ (TBI average: 2.7, SD: 0.1; NC average: 1.8, SD: 0.5, *t*(39) = 3.7, *p* <.001). Participants with TBI also exhibited reduced episodic memory relative to NCs on neuropsychological testing, but there was a range of memory abilities within the groups (Supplementary Digital Content 3, available at: http://links.lww.com/JHTR/B32).

### Feasibility

#### Engagement

All participants completed both sessions in the Blocked schedule, as well as the Training and Delayed Assessments in the Spaced schedule. Participants with TBI completed an average of 9.7 (SD: 0.6) out of 10 possible Retrievals in the Spaced schedule, and NCs completed an average of 9.8 (SD: 0.4) out of 10 possible Retrievals.

#### Technical support

Halfway through the experiment, two participants reported concurrently that MEMI’s image aspect ratio was incompatible with their phone screens. After investigation, the research team determined that a Gorilla update had changed the aspect ratio. We updated MEMI to fit the new parameters, and no further participants reported problems. Fifteen percent of participants (6 total) requested technical support, primarily due to challenges with accessing MEMI due to web browser settings, internet connection, or user error. Each of these participants received support once and completed the rest of the experiment without additional assistance.

### Acceptability

#### Quantitative feedback

Participants in both groups rated both the Spaced and Blocked schedules as acceptable. The TBI group gave a numerically higher acceptability score for the Spaced schedule (16.68, SD: 2.81, very acceptable) than the Blocked schedule (15.31, SD: 4.50, acceptable). In the NC group, the schedule scores were closer (Spaced: 17.55, SD: 2.18, very acceptable; Blocked: 17.25, SD: 2.79, very acceptable).

#### Qualitative feedback

Several themes emerged from participant surveys. Table 1 shows key themes along with representative quotes. Survey questions, more detailed feedback, and participants’ self-reported learning from using MEMI are in Supplementary Digital Content 4, available at: http://links.lww.com/JHTR/B32. When we asked participants how we could improve MEMI, they shared suggestions for branding that will be incorporated prior to a larger trial (more colorful interface, naming the interface rather than calling it “the memory tool” to clarify its purpose).

**TABLE 1:** Participant feedback from free text surveys, by theme. All representative quotes are from participants with TBI. Age is provided in ranges to protect participant privacy

Schedule	Theme	Representative quote
**Spaced**	Noticed progress with frequency and repetition	“Repetitiveness – at first I struggled remembering the words vs. picture and [once] the muscle memory took over it somehow worked.” [male, 26–30]
**Blocked**	Would need more repetitions to be effective	“Was I doing it wrong? I looked at the words/pictures one day and not again for multiple days….What am I missing?” [female, 36–40]
**Both**	Simple and easy to use	“It was consistent and challenged. Not overly complicated.” [female, 36–40]
	Liked incorporation of text messaging	“It was easy to use because it was on my phone.” [female, 46–50]
	Would like to learn personalized information	“I’d like to have the memory tool to use-I hope you make it available. Perhaps something like programming it with things I want to learn or remember or study and scheduling it to quiz me so that I could practice then.” [female, 41–45]
		“I feel I would use a tool like this more so for remembering academic or workplace information that would need to be memorized for a test.” [male, 26–30]

### Preliminary outcomes analysis

The Spaced schedule was associated with more spatial contexts in which participants completed Retrievals (on average, 2.6 contexts in the TBI group, SD: 1.4; 2.9 contexts for NCs, SD: 1.6). The TBI group, on average, remembered fewer words at the Immediate Assessment than the NC group in both the Spaced (TBI average: 4.9, SD: 3.4; NC average: 6.3, SD: 3.5) and Blocked (TBI average: 3.4, SD: 2.4; NC average: 6.5, SD: 3.9) schedules.

**Figure 3.**
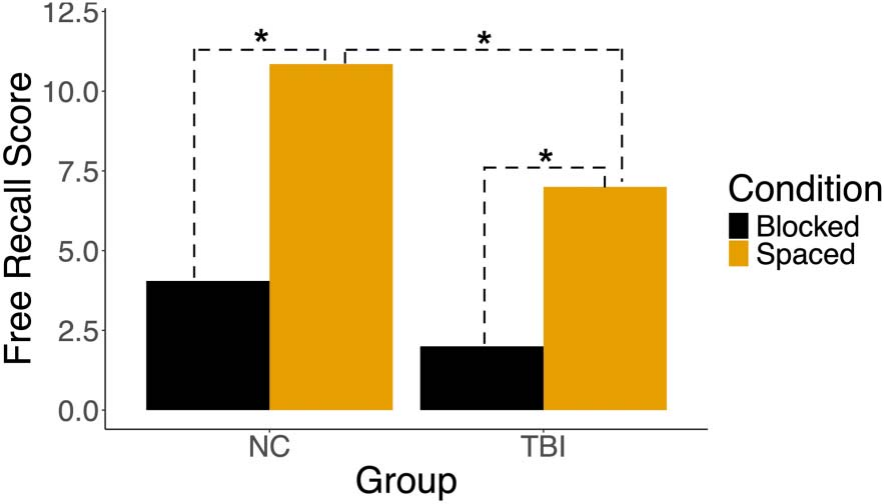
Number of words recalled at the Delayed Assessment, by group and schedule. * = significant at *p* <.05.

**Figure 4.**
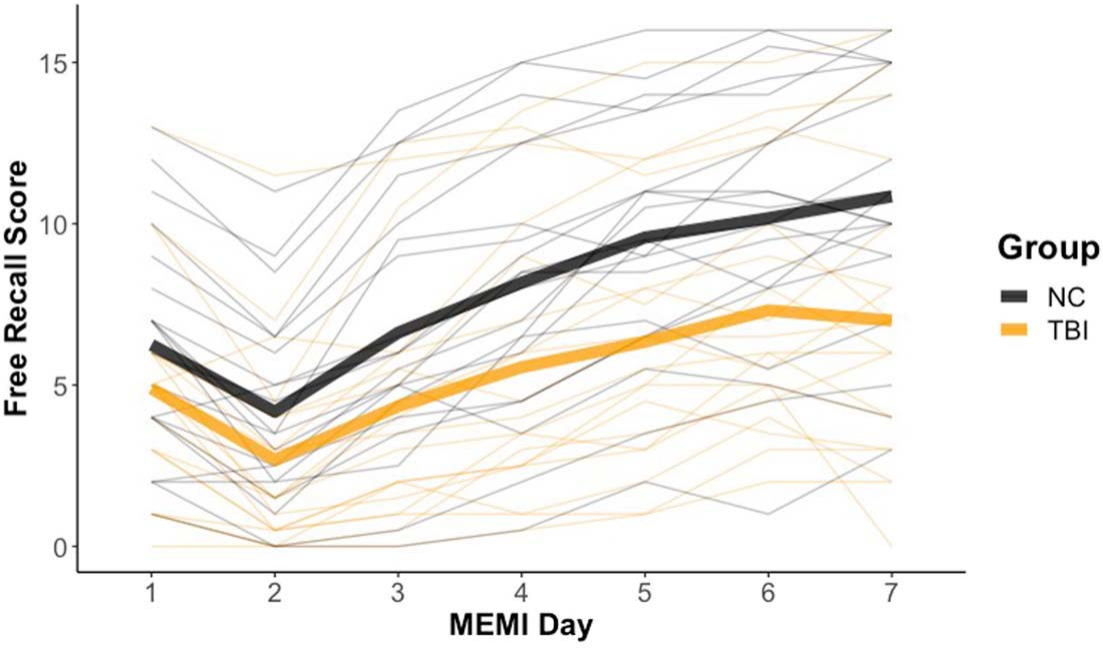
Average free recall performance over the course of the Spaced schedule week (total possible: 16). Thin lines represent individual participants, and bold lines represent group mean performance.

Figure 3 shows free recall performance at the Delayed Assessment by group and schedule. Participants with TBI remembered an average of 5 more words in the Spaced schedule than the Blocked schedule; NCs remembered an average of 6.8 more words. This resulted in a statistically significant difference between the Spaced and Blocked schedules for both groups (TBI: *t*(17) = 4.37, *p* <.001, 95% CI [2.79, 7.99]; NC: *t*(19) = 8.57, *p* <.001, 95% CI [5.13, 8.46]). Of note, the TBI group remembered more words in the Spaced schedule than the NC group did in the Blocked schedule. Figure 4 shows free recall performance over the course of the week, by group, in the Spaced schedule. Although the NC group remembered more words than the TBI group at each timepoint, there was considerable within-group variability, and both groups improved in their recall over the course of the week. We report data on cued recall scores in Supplementary Digital Content 5, available at: http://links.lww.com/JHTR/B32. For cued recall scores, performance mimicked the free recall in that both groups performed better in the Spaced schedule, and adults with TBI remembered more words in the Spaced schedule than the NC group in the Blocked schedule.

## DISCUSSION

### Principal findings

Technology-delivered interventions have potential to extend post-TBI memory care into the chronic phase and daily life. We developed MEMI,^27^ a text message-based memory ecological momentary assessment and intervention system, to support memory using shorter sessions over time and context. In this pilot study, we tested MEMI’s acceptability, feasibility, and intended delivery schedule.

We found that MEMI was acceptable, feasible, and engaging for participants with TBI, replicating our earlier findings^27^ in a larger sample. Participants with TBI found MEMI just as engaging and acceptable as their NC peers. These findings are consistent with and reinforce other work^27,38^ showing that adults with TBI can use and benefit from technology-delivered tools.

Although this was not a fully powered evaluative trial, we found preliminary evidence that MEMI’s intended spaced retrieval schedule is feasible, acceptable, and associated with better long-term recall for adults with TBI. We compared two schedules, one that mimicked a standard single-session rehabilitation schedule (Blocked) and one in which retrievals were spaced over the course of a week (Spaced). The Spaced schedule was associated with better delayed recall in both participant groups, with no corresponding drop in engagement or acceptability. In fact, the TBI group found the Spaced schedule to be more acceptable than the Blocked schedule. The TBI group’s performance in the Spaced schedule was better than the NC group’s performance in the Blocked schedule for both free and cued recall. If these results are replicated in a larger trial, MEMI may help adults with TBI to match non-injured peers on delayed recall of target information.

MEMI may work better for some people than others. Overall, engagement was high, and most participants found the interface to be simple and easy to use. However, three participants (two participants with TBI and one NC) voluntarily withdrew from the study and had diverse reasons for withdrawal. Although the TBI group performed significantly worse than the NC group on canonical neuropsychological memory assessments, the TBI group, on average, performance relatively well. The TBI group also reported more memory failures in daily life than the NC group on the EMQ, a functional memory questionnaire. Canonical neuropsychological memory assessments are limited in their sensitivity and ability to capture key aspects of post-TBI memory deficits (e.g., hippocampal-dependent relational memory^39,40^, memory consolidation deficits that become apparent over time^9^) and do not strongly predict psychosocial outcomes (e.g., community reintegration) as well as memory questionnaires like the EMQ^8^ Thus, they may not predict potential benefit from MEMI. Furthermore, the literature on learning to use systems to address memory impairments suggest that even individuals with severe and selective memory impairment can derive benefits while the absence of other cognitive deficits (e.g., executive function) and the previous use of similar techniques or technology may be strong predictors of success^41–44^. More studies in larger samples are needed to understand how to assess potential MEMI patients and to determine who will benefit most from MEMI.

MEMI has multiple potential roles in a contextualized approach to rehabilitation^17^ that will be assessed in the subsequent evaluative trial. MEMI can bolster a personalized medicine approach to memory rehabilitation by allowing clinicians and patients to identify memory patterns over time. In fact, multiple participants in this study reported noticing patterns in their own memory that may affect their approach to learning in the future. Identifying memory deficits that are disproportionately present at encoding or consolidation may inform rehabilitation approaches for clinicians (e.g., attention-based strategies to support encoding, addressing behavioral modifiers^9,45^ or adjusting timing of learning to support consolidation^9^). Preliminary analyses in this study also suggest that MEMI may support long-term recall of the specific information trained. Because MEMI is expected to improve memory for the trained information rather than memory function more broadly, it is critical that the information in MEMI is personalized to support a contextualized rehabilitation approach. Multiple participants in this study requested personalized MEMI content, especially around academic and vocational goals. For example, MEMI could be used to study for an exam or to learn a new system at work. MEMI could also be used to learn names or facts about people, whether in vocational or personal settings. The personalization of MEMI would require considerable flexibility in the technology. This is an important next step for the tool before progressing to an evaluative trial.

### Next steps

Many participants requested that the target items delivered by MEMI be personalized to their needs and preferences, and this is an important next step to increase ecological validity and clinical utility of this tool. MEMI has many potential uses. In research, MEMI may expand our understanding of how TBI affects memory over time and context, which can lead to new rehabilitation approaches. In the clinic, clients may be able to use MEMI between sessions to support their memory for target items and to help clinicians understand learning patterns to support a personalized medicine approach to memory treatment. Now that MEMI has been determined to be usable, acceptable, and feasible, our next step will be a larger trial of MEMI with personalized information to establish its efficacy^46,47^.

### Study limitations

Although participants were not incentivized for engagement, they were compensated for providing feedback on their experience. Participants may have been reluctant to provide critical feedback due to their ongoing participation in the Brain Injury Patient Registry, but they were encouraged to provide their honest opinions. This participant sample was diverse with regard to sex, age, and education but less diverse with regard to race and ethnicity. To extend the generalizability of findings, the larger trial should include specific considerations for participant diversity in race, ethnicity, and social determinants of health. Some of the survey items do not have psychometric information to report, but composing our own items allowed us to target research questions with face validity.

## CONCLUSIONS

This mixed-methods pilot study revealed that spaced retrieval via MEMI is feasible and engaging for adults with a range of perspectives. It also suggested that spaced retrieval via MEMI may be associated with better long-term recall for adults with TBI, although a larger trial is required for replication and extension of the tool’s clinical utility. This line of work will continue with a larger trial to address the need for flexible, daily memory care in TBI.
